# Visitors’ Perceived Place Value and the Willingness to Pay in an Urban Lake Park

**DOI:** 10.3390/ijerph15112518

**Published:** 2018-11-09

**Authors:** Chanyul Park, Hwasung Song

**Affiliations:** 1 Department of Economics, Hankuk University of Foreign Studies, Seoul 02540, Korea; chan10a@hufs.ac.kr; 2 Department of Finance and Economy, Suwon Research Institute, Suwon 16429, Korea

**Keywords:** place value, economic evaluation, choice experiment, latent profile analysis, urban lake park

## Abstract

As highly developed nature, an urban lake park will be a place required to integrate various functions such as health promotion, recreation, and cultural exchange by focusing on ecological aspects. We applied latent profile analysis (LPA) to identify latent classes based on visitors’ perceived place value, and to estimate the willingness to pay (WTP) by these classifications. Park visitors were classified according to place value into three groups: Local Seekers (LS), Ecology Seekers (ES), and Recreation Seekers (RS). To compare the WTP of the three groups and examine differences in attributes between the groups, we used a choice experiment (CE). The results from the CE revealed that the WTP for attributes was ranked in the order of basic infrastructure, advanced services, and ecological activities. These differences in the WTP of visitors in an urban lake park may be useful for park management, such as providing strategies for zoning and ecotourism, which is specialized by visitor type.

## 1. Introduction

What benefits do people want to gain from a park built in the natural environment? With a growing awareness of the quality of life and the importance of ecological resource management and development, the interest in the value of such resources has grown accordingly. Parks are simultaneously a place for nature appreciation and rest as well as a cultural space for various performances and events. Parks not only provide leisure and entertainment to local residents, but also serve as a tourist destination for non-resident visitors. As such, urban parks offer a wide range of benefits, which are physical, psychological, aesthetic, environmental, economic, social, cultural, historical, recreational, etc. [1,2,3,4,5,6,7], and pursue different core values according to visitors’ perceptions [8,9,10].

In terms of explaining consumer behavior, perceived value is at the top level of human behavioral decisions, and it is an important concept influencing individual attitudes and behaviors [11]. Using the Value‒Attitude‒Behavior (VAB) model from a cognitive hierarchy perspective, Homer and Kahale [12] explain that a value leads to attitude formation, and then to action. In particular, as a value is formed throughout the life of an individual and reflects social cognition, it has the advantage of not changing easily [13]. Thus, value is evaluated as useful for predicting and analyzing user behavior, including willingness to pay [6,11,14,15,16].

Previous research has found that people with higher perceived value have higher rates of satisfaction and intention to revisit [17,18]. Thus, the perceived value has a positive effect on the behavior of users [5]. As services and activities in parks have become more diverse, the values of parks have also become more diverse. Thus, it is essential to understand not only the final behavior of park visitors (i.e., revisiting or recommending) but also the perceived value of the park itself for park management. However, there are not many studies on park and value theories [8,9,10,19] that distinguish the types of park visitors. There are many economic evaluation studies that measure the preference and willingness to pay (WTP) of park visitors [20,21,22,23,24,25,26,27,28,29,30,31]. These studies are largely focused on the conservation of environmental resources and include only a fraction of the WTP for services and activities. However, there are few studies examining empirical evidence of the relationship between the WTP and the perceived value of visitors [5,21,27]. Because these studies estimate the WTP using the contingent valuation method (CVM), they cannot examine which attributes of the park are preferred in the perceived value.

Therefore, the present study will first analyze visitors’ perceived value of an urban lake park and segment the types of visitors based on their perceived value. Specifically, we will use the person-centered approach of Latent Profile Analysis (LPA). Using LPA, visitors can be classified according to their perceived value for parks. Second, we will estimate the WTP using the choice experiments (CE) method. This allows us to compare which attributes the park visitors are more interested in, because a multi-dimensional analysis is possible in CE [32]. Third, we will compare the WTP for attributes by visitor type according to perceived value. This makes it possible to specifically compare how value perception is reflected in actual behavior. The purpose of this study is to find empirical evidence of the relationship between perceived value and WTP, and, by extension, to examine which attributes of the park are preferred according to the types of visitors in an urban lake park. For sustainable development of parks, understanding visitors’ perceived value and preference is very important in managing parks efficiently because of the wide range of park benefits and attributes.

Section 2 explores the previous literature on WTP for parks and perceived place value. Section 3 introduces the methods and materials for analysis including study area, measurement of place value, questionnaire design, data collection, and analysis method. In Section 4, we present the estimation results of the visitor’s WTP and the results of the segmentation, and compare and analyze the differences in WTP by type of visitors. Section 5 and Section 6 present the discussion and conclusions based on the results.

## 2. Literature Review

### 2.1. Perceived Place Value

Place is where physical space is combined with the meaning of human behavioral output, and the perceived value given to this place is called place value [33]. Place value is generated by the place developing over time into a social space that has a unique meaning beyond the physical space [34]. Using the definitions of value types according to function by Sweeney and Soutar [35], place value can be classified into quality value, emotional value, and economic value. Quality value increases when visitors are satisfied with physical service factors such as the environment, ecology, and facilities of a place. The experience of revitalization and enjoyment can be linked to emotional value. Lastly, economic value would be high if visitors are satisfied with the decision to visit, considering the investment of time, effort, and money. Taken together, place value is expressed as the value of putting time and effort into visiting a place, and can be evaluated through the experience and education that can be obtained by visiting a place.

Since Relph [34] divides place-building elements into physical and environmental factors and emotional and cognitive factors, several studies on place values have been done in various places and the results have varied by place and its users. Table 1 summarizes the results of a survey of place value ratings of ecological environmental resources such as national parks, forests, and lakes [8,9,10,19]. The studies include the more traditional place values of ecological environmental resources such as aesthetic/scenic, life-sustaining, and biological diversity values, as well as other diverse values such as intrinsic, spiritual, recreation, and therapeutic value.

Meanwhile, research that typifies visitors based on place value either base the classification on an average place value score or report an exploratory study through qualitative research. As an example of the former, Kaltenborn [36] classified the population of Svalbard, Norway, into groups of high, medium, and low sense of place according to their sense of place index ranking. The limitation of this method is that it overlooks various aspects of place value by using averages. In the case of qualitative research, the study by Hutson et al. [37] that distinguishes types according to the place meanings of natural resources is noteworthy. They measured the place meaning of Canada’s Niagara Escarpment, and classified characteristics according to place value into spirituality seekers, intensity seekers, and sense of self seekers. However, in the case of qualitative research, too, there is the limitation of reliance on subjective evaluation. Latent Profile Analysis is one of the more recent preferred methods for visitor segmentation. LPA is useful for statistically classifying several potential populations and comparing population characteristics [38]. In the present study, we use LPA to segment users’ evaluation of place that is embedded in various aspects of place value.

### 2.2. WTP and Perceived Value of Parks

The economic value of a park includes a variety of non-market values, and generally the method of estimation is based on measures of stated preferences. The representative methods used to estimate the WTP by stated preferences include CVM and CE [32,39]. When the multidimensional approach is needed, CE is preferred over CVM, because attribute-specific WTP can be measured [32]*.* Thus we used CE for a detailed analysis of visitors’ preferences for attributes of an urban lake park. There are many studies on an economic evaluation of parks [1,16,20,21,22,23,24,25,26,27,28,29,30,31,40,41,42,43]. From these previous studies, we were able to see which attributes were most important in order to design the questionnaire for CE.

Prior research on park evaluation using CVM shows that not only the value of the park’s natural environment but also the services and activities associated with the park have an important influence on the WTP [21,22,27,28,29,43]. Reynisdottir et al. [29] compared the WTP of entry fees to the visitors of Gullfoss Waterfall and Skaftafell National Park, major tourist destinations in Iceland, and found that more recreational opportunities lead to higher WTP. Nandagiri [23] evaluated the economic value of water related to recreational use for visitors to Pilikula Lake in India. The results showed that the improvement of lake water quality did not affect the WTP, whereas the benefit of recreation increased the WTP. Most CVM studies are limited, however, in that their analyses are mainly from the perspective of conservation value rather than utilization of environmental resources. Also, due to the limitations of CVM, it is not possible to determine how important the services and activities are in terms of visitors’ preference and perceived value.

In contrast, studies that evaluate the economic value of parks using CE estimate the WTP of each attribute, including the services and activities in parks [1,20,23,30,31,40,41,42]. First, Hearne and Salinas [23] analyzed tourist preferences for nature-based tourism development in Braulio Carrillo National Park in Costa Rica, showing that tourists preferred modern infrastructure and improved views and information. Unlike tourists, the locals were not willing to pay for use restrictions. The WTP of tourists was also much higher than that of the locals. Chaminuka et al. [20] focused on not only environmental resources such as village cultural tours and crafts markets, but also on services and activities with the potential for development of ecotourism, in their examination of preferences for ecotourism in rural communities in visitors to the Kruger National Park in South Africa. As a result, the WTP for accommodation was negative, but the WTP for village tours and crafts markets was positive. Wupper [30] estimated the WTP for tourists in the Jaume Munt National Park in the World Heritage List due to its high ecological value. As a result, not only did the WTP increase according to protection status, fauna, and diversity of habitat, but special attractions and visitor services also had an important influence. Zong [31] examined tourists’ preferences for community-based ecotourism management in Taiwan’s Forest Park. This study also focused on the WTP for various services such as tour guide interpretation, travel information, accommodation style, and experience activity, rather than for the value of natural resources. In line with previous studies, we selected the attributes for CE, with a focus on utilization of environmental resources, as well as services and activities. Furthermore, we examine which attributes are preferred according to the segmentation based on visitors’ perceived value.

There is some quantitative research that has discussed the effects of psychological preference on WTP [1,16,21,22,24,26,27]. Hearth and Kennedy [24] found that only payment attitude among the socioeconomic variables had a significant effect on the WTP. Kmakawa [26] found that the higher the pro-social behavior and trust, the more significant and positive the impacts were on the WTP for the preservation of scenic lake landscapes. López-Mosquera and Sánchez explored the effect of psychological variables on WTP. They found that positive emotion and satisfaction were the determining factors of the WTP for the improvement in the conservation of natural areas [27]. Cheung et al. [21] focused on understanding visitors’ preferences and the WTP for geoparks. They categorized visitors’ preferences into two groups—intrinsic value and extrinsic facilities—using cluster analysis based on perceived value. Moreover, more than half of the preferences included had significant positive correlations with the WTP estimated by CVM. Cheung and Jim [22] focused on the differences between nature tourists and general tourists, and found significant correlations between the WTP and expectation of ecotourism services. Henderson-Wilson et al. [5] argued that higher WTP is correlated with a higher perceived value placed on exercising, socializing, and relaxing in the park.

Since the psychological preferences examined in the previous studies determine the satisfaction, benefit, and WTP, they are very similar to the perceived values we are interested in. However, as prior studies focused on the significance of the correlations between values and the WTP, it is not possible to evaluate the differences in the WTP according to the perceived values of park visitors.

## 3. Method and Materials

### 3.1. Study Area

**Figure 1:**
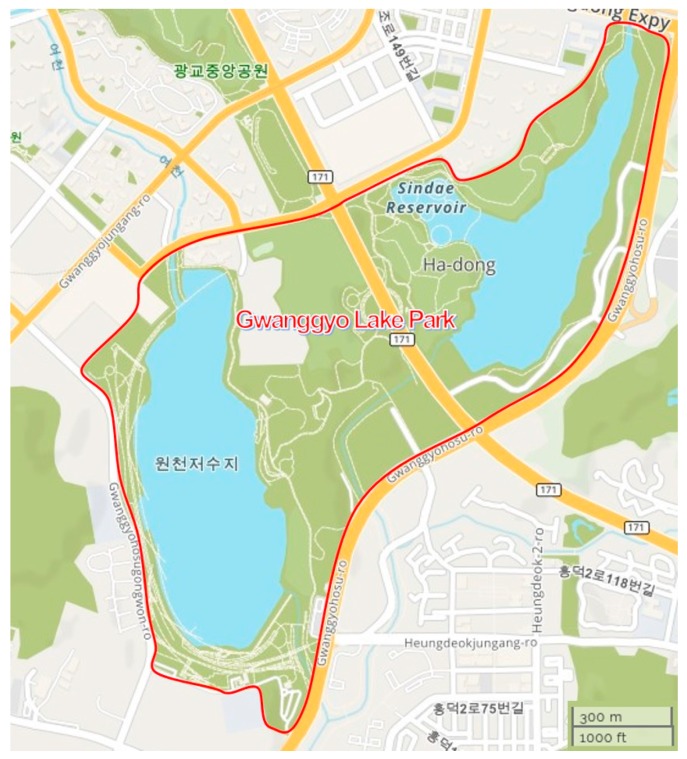
Map of Gwanggyo Lake Park.

The multiple characteristics of Suwon’s Gwanggyo Lake Park (GLP), as an urban lake park promoting rest and health, an urban neighborhood park, and waterside ecotourism resource, mean that it attracts diverse types of visitors (see Figure 1). For this reason, GLP provides a research location that is well suited for the present study.

GLP is the largest lakeside park in Korea, established by integrating the Wonchon recreation area and Shindae Lake in 2013. GLP is evaluated as having high ecological and aesthetic value due to its high preservation of lakes and forests, and is used as a location to experience and learn about the ecological environment. It also offers various programs and events including nature ecology, traditional culture, art, and citizen participation. There are nature-based tourism resources of lakes and forests, experiential activities, festivals, and events, as well as camping grounds, within the park. In addition, GLP is located in Suwon City, a historical and cultural city represented by Hwaseong Fortress, a UNESCO World Heritage site, and one of the cities in Korea with high tourism demand. Therefore, GLP is not just an ecological and environmental park, but contains various values due to its connections with diverse services and activities in nearby leisure sites. Considering China’s Summer Palace and Wisconsin Lake in the USA, which are used both as a place of rest and exercise and as a cultural/recreation resource for local residents, GLP, which provides comparable facilities and programs, has the potential to be designated as Korea’s representative recreation park.

### 3.2. The Measurement of Values and Attributes

Sweeney and Soutar [35] classified value into quality value, emotional value, and economic value. The ecological, cultural, and economic values of natural resources continue to be measured, according to Brown [9] (see Table 1). In the present study, as economic value was estimated using a CE, the economic value items were excluded from place value measurement. Instead, we added the residential and local activity participative values, taking into account the locational characteristics of the GLP. The value of GLP was judged to be different from that of other tourist attractions, as it is the place where an amusement park was originally located, it is a waterside recreation park with a special place value that reminds visitors of past memories, and it is valued by the local residents who have lived in the area for a long time.

For the CE, the attributes and levels of GLP were employed based on previous studies and similar cases. As stated in the literature review, the WTP for ecological environmental resources is determined not only by the basic natural environment and infrastructure, but also by the services and ecological activities that are provided [20,23,30,31]. Therefore, the attributes of GLP were composed of basic infrastructure, advanced services, and ecological activities (see Table 2). In order to set attribute levels, a similar urban lake park example was examined and the following hypothetical scenarios were assumed.

- Scenario I—Maintain current basic infrastructure, services, and activities (Level 1).
- Scenario II—Supplement additional infrastructure, services, and activities (Level 2).
- Scenario III—Develop the ideal infrastructure, services, and activities (Level 3).

Scenario I assumes the status quo of GLP infrastructure, services and activities provided, and corresponds to Level 1, which is the baseline comparison group. Scenario II refers to some additional services and activities that can be improved and provided within the park. Scenario III includes ideal conditions such as improvement efforts and investments, and connections with other leisure sites outside the park. We also conducted interviews and discussions with experts in outdoor recreation, landscape, and economics in order to make adjustments to the attributes and levels.

Each attribute consists of Levels 1–3 according to the hypothetical scenarios. Level 1 of the first attribute, basic infrastructure, includes basic rest space that is provided mainly as part of the landscape, such as benches and shade. Level 2 provides sufficient trails and bike paths for more active enjoyment of lake scenery. Level 3 is the expansion of various exercise facilities to increase utilization such as health enhancement, as well as to improve the infrastructure to maximize the beauty of the lake landscape.

Level 1 of the second attribute, advanced services, indicates the installation and managing of the park’s basic service facilities such as restrooms and shops. Level 2 is to introduce additional services in the park to increase the convenience of park users such as additional public parking lots, services and facilities for the elderly and disabled persons, and limitations to prevent crowding and accidents. Level 3 includes improvements to service, such as transportation, and campground and guide interpretation, to maximize ecotourism convenience.

Level 1 of the third attribute, ecological activities, is the provision of basic sights and attractions, including temporary events revolving around landscaping. Level 2 provides additional ecological experience and learning programs, and various waterside activities that allow for more active use of ecological resources. Level 3 is the provision of leisure activities based on ecological resources within the park, as well as ecotourism packages that include connections to nearby leisure sites.

### 3.3. Questionnaire Design

For the choice experiment, a choice set based on the level-specific combination of attributes from Table 2 was constructed. In a full factorial design of the four attributes including Payment, 108 (3^3^ × 4) profiles are derived. Since it is impossible to evaluate all of them, a fractional factorial orthogonal design is preferred in general [32,44]. Thus, the choice set for the experiment only included 12 profiles derived by an orthogonal design using SPSS Version 23 (IBM, Armonk, NY, USA). Respondents randomly chose three of the 12 profiles, and selected the preferred profile among them, including the status quo without payment. The example of a randomly selected choice card is on the Table 3. The experiment was performed five times for every respondent. Valid responses were retrieved from 652 respondents, and the experiment was conducted a total of 3260 times. Based on the difficulty of collecting admission fees to GLP, we used the taxes for the payment vehicle. The respondents chose the preferred profile including payment level, which means how much more they would pay in taxes for park improvement, maintenance, and management.

### 3.4. Data Collection and Analysis

The target population of this study includes visitors to GLP of both non-resident tourists (i.e., outsiders) and local Suwon residents, as GLP was used as a representative place for leisure and tourism resources in South Korea. The pre-test was first administrated to 10 visitors prior to the main survey. Based on the results and feedback from the pre-test, the ambiguous items were revised. The main survey was conducted for 16 days on weekends and weekdays in May 2017 at designated entry/access points and public rest sites in GLP using on-site interviews. The random sampling strategy used to select interviewees at GLP was based on a systematic approach. It involved determining (a) the number of individuals to be sampled each day and (b) how many individuals should be sampled per hour. Interviewers based their selection on the number of arrivals of users to the site. Interviewers approached every third visitor/group encountered at GLP. The questionnaire was self-administrated and took approximately 15 min to complete. Out of 700 questionnaires received, we used 652 samples for the data analysis, excluding invalid responses. The questionnaire included CE questions, socio-demographic characteristics, park use status, recognition of park value, and intention to revisit. The respondents were primarily female (58.7%), in their 40s (30.8%), local residents (32.1%), and visited 2–3 times per month (35.6%).

A CE defines the indirect utility function through the choice of respondents based on a random utility model [45]. McFadden [45] presented the measurement of the indirect utility function through the irrelevant independence alternative (IIA) and conditional logit model (CL). Consistent with previous evaluation studies of parks [20,23], the indirect utility function of visitors was estimated using the CL model, assuming IIA, in the present study. Using a maximum likelihood estimator of attribute levels, the marginal willingness to pay (MWTP) for attribute levels can be calculated. This leads to an estimation of all visitors’ preference for GLP, that is, the MWTP for each attribute level. Since the variables for attribute level are dummy variables, the estimated MWTP indicates differences from the baseline (Level 1).

Meanwhile, Latent Group Analysis (LGA) is a type of mixture model analysis that identifies subgroups, that is, latent groups within a sample, to identify diversity within a population [46]. LGA has the advantage of determining group classification according to statistical criteria including fit indices, entropy, LMR, and LRT, instead of using cluster analysis or the subjective interpretation of the researcher [47]. That is, LGA is not based on an arbitrary standard score, but based on sample-specific characteristics related to the variables of interest to the researchers. LGA is divided into Latent Class Analysis (LCA) and LPA. Whereas LCA is used for exogenous variables measured as binary (categorical) and nominal variables, LPA is used with continuous variables [48]. Compared to discrete data, continuous data can give a variety of analysis options that can offer insight into the source of variation. In particular, as the concept of value is more abstract and higher rank than attitude and behavior, it is measured as a continuous variable rather than discrete data [49]. Thus, we used LPA to capture the perception of place value of GLP visitors. In addition, LPA results were used to estimate the choice experiment results for each visitor type through the CL model, and to compare differences among groups using calculated MWTP for attribute levels.

## 4. Results

### 4.1. Willingness to Pay in GLP

Table 4 shows the CL model estimates and the MWTP of the total visitors in GLP, with 90% confidence intervals using Krinsky Robb’s parametric bootstrapping [50]. The coefficient estimates of PAY were negatively significant (*p* < 0.01). As the payment amount increased on the choice card, the probability of selection decreased, indicating rational decision-making had taken place. All estimated coefficients of attributes were positively significant, indicating that visitors of GLP preferred higher levels of attributes compared to the status quo.

Using the CL model estimation results, we can obtain the MWTP, an additional payment intention relative to the status quo (Level 1), for each attribute. In order to compare visitors’ WTP for the highest level of attributes, the differences in MWTP between Level 1 and Level 3 were in the descending order of basic infrastructure (18,261 KRW), advanced services (13,965 KRW) and ecological activities (12,683 KRW) (BI3 > AS3 > EA3). Visitors were most willing to pay for the expansion of various exercise facilities and the improvement of infrastructure and landscaping The MWTP for advanced services and ecological activities was also positive but relatively lower than that of basic infrastructure.

Since Level 3 included the materials of Level 2, the differences between Level 2 and Level 3 indicated the MWTP for Level 3 compared with Level 2. For basic infrastructure and advanced services, the MWTPs increased consistently with increasing level, but not for ecological activities. In the case of ecological activities, the 90% confidence intervals of the MWTP for Level 2 overlapped with that of Level 3, indicating that they were not different at the 10% significance level. This is due to visitors’ low interest in connections to nearby leisure sites at Level 3 of ecological activities. However, this may vary depending on the type of visitor, and a detailed discussion can be found in the analysis of visitor type in Section 4.3.

### 4.2. Segmentation of GLP Visitors Based on Perceived Values

LPA was administered to identify the latent subgroups of visitors based on the 11 items of place value, which indicate visitors’ perceived value of GLP. Fit indices (i.e., AIC, BIC), entropy (i.e., quality of classification), and LMR LRT (i.e., Log likelihood comparison in k and k-1 model) were used to compare models with two to three latent profiles (Table 5). However, despite the usefulness of these indices, there are no absolute fit criteria in deciding the number of profile [47] because it is also important to consider the existence of diverse patterns, and the theoretical and practical interpretability of the selected profiles.

In Table 5, the three-profile model displayed the lowest values of AIC (19,146.67) and BIC (19,406.51), but it was not statistically significant in LMR LRT. The three-profile model showed relatively lower values of AIC (19,454.55) and BIC (19,660.631) with the entropy value (0.897) close to 1, and was significant (*p* < 0.01). The two-profile model was also significant, but AIC, BIC, and entropy were not better than in the three-profile model. Thus the three-profile model was selected as the optimal.

**Figure 2:**
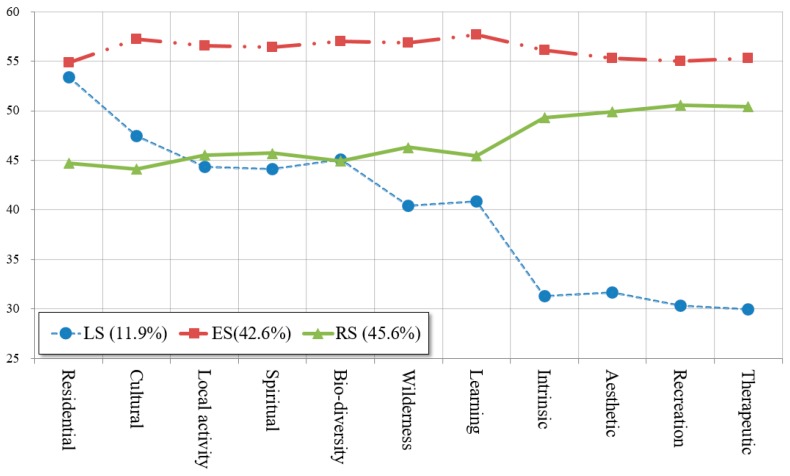
*T*-scores of place value for each profile.

Table 6 summarizes the three latent profiles of 652 visitors based on place value. It shows the average scores of the 11 items of place value rated on 5-point scales. The overall average score was 3.41, with the highest average score for therapeutic value (M = 4.29) and the lowest average score for cultural value (M = 3.23) among all profiles. For a clear comparison of the differences between profiles, the results were converted to *t*-scores, which are standardized by items to *N* (50,10) and presented in Figure 2.

The characteristics of each profile were as follows. The first profile (11.2%, *n* = 78) displayed an average score of 2.56. Most of the scores in this profile were the lowest overall among the groups, while the residence, cultural, and local activity participative values were higher. This profile indicated a high preference for “locality” and was thus named the “Local Seeker” (LS) group. LS included the highest proportion of local residents (64.1%), and 2–3 visits per month (44.9%) among the three profiles.

The second profile (42.6%, *n* = 278) indicated the highest level of place value in all the response items with an average score of 4.37. In particular, this profile scored relatively higher on ecological values such as the learning, biological diversity, and wilderness values. In the present study, learning value is concerned with the utilization of a place to learn from the environment. As this profile placed high value on the ecological value of nature itself, this profile was labeled the “Ecology Seeker” (ES) group. The percentage of local residents in this group was 60.1%, and had the highest proportion of 2–3 visits per week (39.6%) among the three profiles.

The third profile (45.6%, *n* = 297) showed an average score of 3.30. It displayed the middle ranking in almost all of the items among the groups. This group showed a higher preference for the recreational aspects of nature, such as the aesthetic, recreation, and therapeutic value. They seem to feel better physically and mentally through the scenery and outdoor recreation activities in the environment. Thus, this profile was labeled the “Recreation Seekers” (RS). Compared with the other two profiles, the proportion of local residents (54.8%) was relatively lower, indicating the highest proportion of non-residents among the three groups. Also, the proportion of first-time visiting or once or twice a year (41.5%) was highest in this group.

Although ES showed the highest scores in all of the place value items, the results indicated varying levels of place value across the three profiles. The LS had the lowest scores for recreation value and relatively higher scores for locality value, and a reverse of this pattern was found for the RS. This finding implies that researchers may need to use an idiographic approach (i.e., a person-centered approach) to examine the concept of place value. In this sense, LPA was an adequate and effective method to identify the various aspects of place value of GLP visitors.

### 4.3. Willingness to Pay and Types of Visitors

Table 7 shows the estimated results of CL model estimates from the CE by the type of visitor in GLP. Every coefficient of attribute levels was positively significant at the 1% level except for the advanced services Level 2 (AS2) for LS. Particularly, in the case of LS, the estimation coefficient of advanced services Level 2 (AS2) was not significant even at the 10% level.

Table 8 shows the calculated MWTP from a CL estimation with 90% confidence intervals by type of visitor. When MWTP was calculated for the attributes and levels by group, a comparison of the differences between attribute levels within group, and the differences between group within the attribute levels, revealed the following main findings.

First, in all three groups, the MWTP was positive for all attribute levels and highest for basic infrastructure (BI3) including improved exercise facilities and landscaping. However, the second highest attribute level was advanced services (AS3) for RS in contrast to LS and ES. This means visitors’ WTP and the magnitude of needs for attributes differ by the type of visitor, that is, the perception of place value of the park. Thus, these differences should be considered in park management, especially in those with diverse values, such as GLP, an urban lake park.

Second, between the three groups, LS had the lowest MWTP. However, the differences between the groups were not statistically significant at the 10% level, except for the MWTP for advanced services Level 2 (AS2). The MWTP for advanced services Level 2 (AS2) was significantly lower for LS when compared to ES, but not to RS. This is because the LS group, which emphasized local culture and activities, had the highest percentage of local residents, and thus did not feel the need for the expanding of public parking lots or limiting the use of bicycles. This is in line with Hearne and Salinas’ findings that tourists showed a strong preference for restrictions on access, whereas local residents did not [9]. In addition, the WTP of local residents was lower than that of outsiders, indicating different perceptions in the use and value of parks. In the present study, the LS had the lowest WTP for attributes, because they only considered the value of local places such as friends, relatives’ residence, and local cultural activities. Therefore, interest in environment development, services and activities, and overall MWTP were very low. The highest MWTP was seen in RS for the basic infrastructure and advanced services attributes, and in ES for the ecological activities attribute. RS who perceived place value based on recreation and leisure, had a high level of MWTP, and had relatively higher WTP for infrastructure and services for outdoor recreation and leisure. Meanwhile, the ES showed higher WTP for ecological experience and learning programs, various leisure activities, and ecotourism packages that make greater use of ecological resources.

Third, as the estimation results for the entire sample of visitors, the MWTPs increased consistently with increasing level for basic infrastructure and advanced services, but not for ecological activities. In the MWTP for ecological activities, the subtraction of Level 2 from Level 3 was only positive for ES, in contrast to LS and RS. This shows that only ES had a positive MWTP for the additional materials of ecological activities Level 3, including leisure activities based on ecological resources within the park, as well as ecotourism packages including connections to nearby leisure sites. Thus, for ES, it would be worthy to not only develop ecological resources for leisure in the park, but also expand ecotourism that involves collaboration with surrounding heritage and tourism sites.

## 5. Discussion

The results showed that GLP visitors’ preference for the highest level of attributes was in the order of basic infrastructure, advanced services, and ecological activities. Thus, visitors are most willing to pay for infrastructure including improvement of the lake landscape, and more facilities for health promotion, etc. Each MWTP for attributes of the park was positively significant, which is consistent with previous findings [20,23,30,31]. Whereas previous studies have estimated WTP only for some of the park infrastructure and services and activities, the present study extends this literature by comparing WTP while including both of these at the same time.

Following the recommendation by Brown [8], the present study expanded the scope of perceived place value research to a broader population. Whereas previous research mainly measured place value in semi-developed nature (i.e., forests, lakes, national parks targeting nature value itself), the present study focused on highly-developed nature (i.e., an urban lake park). Also, whereas previous research focused on the place value of ecological environmental resources in terms of aesthetic/scenic, biological diversity, therapeutic, and recreation values [8,9,10,19], the present study placed emphasis on the cultural exchange and residential functions characteristic of an urban lake park, by including residential, culture, and local activity participative values to the locality value. As a result, visitors of highly developed natural places were classified as LS, ES, and RS. In contrast to Kaltenborn’s study [36], which classified upper, middle, and lower groups based on the overall average of place value, LPA yielded groups that reflect the diversity in place value. The qualitative work on various place values by Hutson et al. [37] was further extended through the present quantitative research. In particular, whereas Brown and Raymond [9] and Zhu et al. [19] evaluated place value by dividing their sample into local and non-residents, the present study segmented the sample based on place value, and then examined residence by group. For example, the RS is a group that seeks the recreational use of natural resources, with a high proportion of non-residents, whereas the LS is a locality-seeking group that has a high proportion of residents. The findings of the ES with high environmental concern indicate that more diverse group segmentation is possible. LPA was particularly useful in examining the multiple aspects of place value.

The CE model for each group of visitors was estimated and compared, which yielded several differences. This approach extends the findings of previous studies by showing the differences, not only the correlations [21,22,27], according to perceived value. It also improves upon previous studies, which assumed heterogeneous visitors and classified using socio-demographic characteristics [1,23,31,42,51,52], by segmenting the visitors into groups using perceived value and examining differences between groups. As a result, the most preferred attribute varied depending on how place value was perceived. Whereas the LS had the lowest MWTP in general, similar to previous research showing that greater recreational opportunities had a significant effect on visitor’s WTP [20,28,29,30,31], RS, for which recreational value had the highest importance, mostly showed the highest MWTP. Thus, beyond basic infrastructure, improving services and activities that promote recreational utility for visitors is also important. In addition, ES who recognized the place value associated with ecological resources and programs, displayed a positive WTP for the inclusion of connections to nearby leisure sites. This points to the possibility of extensions to ecotourism for sustainable usage and ecological development of parks based on natural environments such as GLP, similar to the findings of Wupper [30] and Zong [31].

## 6. Conclusions

The purpose of this study was to examine which attributes of the park are preferred by different visitor types, which was determined by perceived place value in an urban lake park. A park inherently holds diverse functions as a place providing beautiful scenery and resting space, and a cultural hub in which various performances and events are held. Its functions and visitors’ benefits also vary according to the characteristics and the status of the park.

In the present study, we estimated the WTP by the type of visitor to the GLP, an urban lake park. GLP, the subject of this study, is the largest of the lake parks in Korea and is a recreational lake park providing health promotion, outdoor activities, and festivals and events to visitors. It has the potential to be designated as Korea’s representative recreation park, similar to China’s Summer Palace and Wisconsin Lake in the U.S. Thus, to examine the WTP including services and activities based on ecological resources in GLP, the attributes of park value were designated as basic infrastructure, advanced services, and ecological activities.

The main contribution of this study is that we confirm the differences of the WTP by visitor type in an urban lake park, which provides useful implications for park management. For instance, the park manager is able to make zoning strategies and introduce various ecotourism packages specialized by visitor type. To elevate the WTP of local residents, furthermore, it is necessary to changes of visitors’ perceived values for more visitors in ES and RS than LS.

There are some limitations of present study. Since we used taxes for the payment vehicle instead of admission fees, the results could be underestimated by the payment vehicle bias. We did not subdivide the attributes more specifically due to the interest in comparing the WTP by visitor type in an urban lake park. In future research, it will be necessary to look at a more direct alternative for sustainable usage and ecological development of lake parks by subdividing the attributes more specifically according to visitors’ purposes in park use. In addition, based on the finding of a positive WTP for the ecotourism package in the highest ecological concern group, virtual ecotourism packages should be created and WTP for these packages determined, so that improvement plans for ecotourism activation can be presented.

## Figures and Tables

**Table 1:** The measurement of place value in natural resources.

Value	Brown [1]	Brown & Raymond [2]	Beverly et al. [3]	Zhu et al. [4]
Aesthetic/Scenic value	◎	◎	◎	◎
Economic value	◎	◎	◎	◎
Recreation value	◎	◎	◎	◎
Life Sustaining value	◎	◎		◎
Learning value	◎	◎	◎	◎
Biological diversity value	◎	◎	◎	◎
Spiritual value	◎	◎	◎	◎
Intrinsic value	◎	◎		◎
Historic value	◎		◎	
Future value	◎	◎		◎
Subsistence value	◎		◎	
Therapeutic value	◎	◎		◎
Cultural value	◎			
Wilderness value	◎	◎	◎	◎
Heritage value		◎		◎
Existence Value			◎	

◎ means ‘it is included’.

**Table 2:** The attributes and levels for choice experiment.

Attributes	Levels	Variable Name ^1^
Basic Infrastructure	1. Basic seating areas	BI1
	2. Additional trails and bike paths	BI2
	3. Improvements on infrastructure and scenic views	BI3
Advanced Services	1. Basic service facilities	AS1
	2. Additional service facilities	AS2
	3. Improvements on service facilities including transportation, campground, and guide interpretation	AS3
Ecological Activities	1. Basic sights and events	EA1
	2. Ecological experience and learning, various waterside activities	EA2
	3. Additional attractions and activities including connections to nearby leisure sites	EA3
Payment	1. 5000 KRW per household and year	PAY
	2. 10,000 KRW per household and year	
	3. 20,000 KRW per household and year	
	4. 30,000 KRW per household and year	

^1^ Each variable is a dummy variable, except for PAY.

**Table 3:** Example of a randomly selected choice card.

Attributes	Alternatives			
	Card 1	Card 2	Card 3	Card 4
Basic Infrastructure	1. Basic seating areas	1. Basic seating areas	3. Improvements on infrastructure and scenic views	2. Additional trails and bike paths
Advanced Services	1. Basic service facilities	2. Additional service facilities	1. Basic service facilities	2. Additional service facilities
Ecological Activities	1. Basic sights and events	2. Ecological experience and learning	2. Ecological experience and learning	1. Basic sights and events
Payment	0 KRW	30,000 KRW	10,000 KRW	5000 KRW

**Table 4:** Estimated results and MWTP (marginal willingness to pay) of visitors in GLP.

Attribute and Level		Coefficient ^1^	Standard Error	MWTP ^2^	90% CI of MWTP ^3^	
					Lower Bound	Upper Bound
PAY		−5.5 × 10^−5^ ***	2.54 × 10^−6^	-	-	-
Basic Infrastructure	BI2	0.585 ***	0.053	10,669	9016	12,259
	BI3	1.002 ***	0.052	18,261	16,720	19,838
Advanced Services	AS2	0.507 ***	0.052	9252	7684	10,882
	AS3	0.766 ***	0.052	13,965	12,348	15,632
Ecological Activities	EA2	0.726 ***	0.053	13,235	11,670	14,909
	EA3	0.696 ***	0.055	12,683	11,231	14,256
Number of observations = 12,949			Log likelihood = −4092.9			

^1^ Coefficients are the maximum likelihood estimators of the conditional logit model (*** *p* < 0.01). ^2^ MWTP is the marginal willingness to pay that is measured by KRW per household and year compared with the status quo (Level 1). ^3^ The 90% confidence intervals (CI) are calculated from parametric bootstrapping using Krinsky Robb’s method [50] in STATA 13.

**Table 5:** Latent profile model fit indices of place value.

Number of Classes (*k*)	AIC	BIC	Entropy	LMR LRT *p* Value	Adjusted LMR LRT *p* Value
2	20,660.100	20,812.422	0.867	<0.0001	<0.0001
3	19,454.549	19,660.631	0.897	<0.0001	<0.0001
4	19,146.667	19,406.510	0.859	0.0786	0.0790

AIC = Akaike information criterion; BIC = Bayesian information criterion; LMR = Lo‒Mendell‒Rubin; LRT = Likelihood Radio Test (comparison with a (k-1) class model).

**Table 6:** Sample characteristic by profile.

Items	Local Seeker; LS (11.9%)	Ecology Seeker; ES (42.6%)	Recreation Seeker; RS (45.6%)
Residential value	3.40	3.61	2.17
Cultural value	2.92	4.09	2.52
Local activity participative value	2.69	4.14	2.84
Spiritual value	2.64	4.21	2.85
Biological diversity value	2.87	4.16	2.86
Wilderness value	2.62	4.38	3.25
Learning value (knowledge)	2.65	4.51	3.16
Intrinsic value	2.08	4.82	4.07
Aesthetic value	2.06	4.67	4.07
Recreation value	1.99	4.67	4.19
Therapeutic value	2.19	4.84	4.33
Average	2.56	4.37	3.30
*N*	78	278	297

Note: Rated on a 5-point scale from 1 (strongly disagree) to 5 (strongly agree).

**Table 7:** Estimated results by type of visitor.

Attribute and Level		Type of Visitors		
		Local Seeker	Ecological Seeker	Recreational Seeker
PAY		−5.9 × 10^−5^ *** (7.6 × 10^−6^)	−5.3 × 10^−5^ *** (3.9 × 10^−6^)	−5.6 × 10^−5^ *** (3.8 × 10^−6^)
Basic Infrastructure	BI2	0.594 *** (0.154)	0.530 *** (0.082)	0.646 *** (0.079)
	BI3	0.824 *** (0.159)	0.943 *** (0.080)	1.105 *** (0.077)
Advanced Services	AS2	0.174 (0.157)	0.570 *** (0.077)	0.532 *** (0.078)
	AS3	0.648 *** (0.145)	0.746 *** (0.081)	0.827 *** (0.077)
Ecological Activities	EA2	0.657 *** (0.154)	0.714 *** (0.082)	0.758 *** (0.077)
	EA3	0.579 *** (0.150)	0.810 *** (0.085)	0.625 *** (0.081)
Log likelihood		−491.6	−1723.0	−1868.7
Number of observations		1540	5423	5986

Note: Coefficients are the maximum likelihood estimators of the conditional logit model (*** *p* < 0.01), and the standard errors are in parenthesis.

**Table 8:** The comparison of MWTP by type of visitor.

Attribute and Level		Type of Visitors		
		Local Seeker	Ecological Seeker	Recreational Seeker
Basic Infrastructure	BI2	10,111	10,078	11,466
		[5838, 14,328]	[7413, 12,670]	[9079, 13,784]
	BI3	14,031	17,947	19,611
		[10,093, 18,046]	[15,454, 20,531]	[17,392, 21,999]
Advanced Services	AS2	2968	10,842	9440
		[−1540, 7429]	[8352, 13,457]	[7149, 11,836]
	AS3	11,038	14,185	14,677
		[6918, 15,303]	[11,541, 17,020]	[12,363, 17,176]
Ecological Activities	EA2	11,200	13,580	13,460
		[7288, 15,846]	[11,070, 16,283]	[11,263, 15,855]
	EA3	9862	15,407	11,099
		[6135, 14,142]	[13,071, 18,070]	[9065, 13,396]

Note: The marginal willingness to pay (MWTP) is calculated from the estimated coefficients of a conditional logit model and measured by KRW per household and year compared with the status quo (Level 1) of each attribute. The 90% confidence intervals (CI) are in brackets and calculated from parametric bootstrapping using Krinsky Robb’s [50] method in STATA 13.
